# Ethical dilemmas due to the Covid-19 pandemic

**DOI:** 10.1186/s13613-020-00702-7

**Published:** 2020-06-17

**Authors:** René Robert, Nancy Kentish-Barnes, Alexandre Boyer, Alexandra Laurent, Elie Azoulay, Jean Reignier

**Affiliations:** 1 Université de Poitiers, Poitiers, France; 2 Inserm CIC 1402, Axe Alive, Poitiers, France; 3 Service de Médecine Intensive Réanimation, CHU Poitiers, Poitiers, France; 4 Service de Réanimation Médicale, APHP, CHU Saint-Louis, Paris, France; 5 Groupe de Recherche Famiréa, Paris, France; 6 Université de Bordeaux, Bordeaux, France; 7 Service de Médecine Intensive Réanimation, CHU Bordeaux, Bordeaux, France; 8 Laboratoire psy-DREPI, Université de Bourgogne Franche-Comté, 7458 Dijon, France; 9 Service de Réanimation Chirurgicale, Dijon, France; 10 Université de Nantes, Nantes, France; 11 Service de Médecine Intensive Réanimation, CHU de Nantes, Nantes, France

**Keywords:** Covid-19, Pandemic, ICU, Ethics, Triage, Withdrawal of life support, End-of-life, Family-centered care, Burnout

## Abstract

The devastating pandemic that has stricken the worldwide population induced an unprecedented influx of patients in ICUs, raising ethical concerns not only surrounding triage and withdrawal of life support decisions, but also regarding family visits and quality of end-of-life support. These ingredients are liable to shake up our ethical principles, sharpen our ethical dilemmas, and lead to situations of major caregiver sufferings. Proposals have been made to rationalize triage policies in conjunction with ethical justifications. However, whatever the angle of approach, imbalance between utilitarian and individual ethics leads to unsolvable discomforts that caregivers will need to overcome. With this in mind, we aimed to point out some critical ethical choices with which ICU caregivers have been confronted during the Covid-19 pandemic and to underline their limits. The formalized strategies integrating the relevant tools of ethical reflection were disseminated without deviating from usual practices, leaving to intensivists the ultimate choice of decision.

## Background

In their daily practice, intensivists are used to facing to ethical concerns related to admission or non-admission to ICU, to withholding or withdrawing life support and to communication with families. The devastating pandemic that has stricken the worldwide population induced an unprecedented influx of severe ARDS patients dramatically exceeding ICU bed capacities in several areas of many countries. As a result, four new options never applied to date were considered with the common aim of saving a maximum number of lives: to prioritize ICU beds for patients with the best prognosis; to increase at all costs the number of ICU beds, thereby creating step-down ICUs; to organize transfer to distant ICUs with more beds available, or to accelerate withdrawal of life support in ICUs. Additionally, to protect the patients’ relatives, visits for families were prohibited or strongly limited and adequate communication between caregivers and families was disrupted, counteracting more than 20 years of research aimed at improving interaction with families and quality of care during EOL [1]. Moreover, since most health care facilities were being used for Covid-19 patients, the situation also raised concerns inside the ICU for patients without Covid-19 requiring ICU admission.

In such a crisis, there are ingredients liable to shake up our ethical principles, sharpen our ethical dilemmas, and lead to situations of suffering for caregivers [2]. Faced with these profound changes in patient management, intensivists were caught off guard, forced by the density of work, the lack of immediately available beds and the possibilities of transferring patients to make painfully experienced choices that were contrary to their basic ethical principles and source of immediate burden [3–5]. The aim of this paper is to focus on and to discuss the main ethical concerns raised during the pandemic, especially with regard to ICUs. Since there are differences between health organizations in different countries around the world, ethical perception may vary according to legal or societal specificities. However, even though our thinking was based on French management of the crisis, similar approaches were assessed in other countries, especially in Europe and ethical questioning is commonly shared by intensivists throughout the world.

## Main text

### Modification of admission or non-admission strategies (triage)

The massive influx of patients raised questions on the eventual modification of our admission criteria to the detriment of the most vulnerable populations.

The decision to refuse admission of a severely ill patient to an ICU is a regular part of the intensivist’s work. Guidelines have been drawn up to guarantee fairness, avoid unreasonable obstinacy and ensure respect for the patient’s wishes and transparency with families [6]. Theoretically, even during an epidemic ICU patient admission decision-making should be identical to that of a routinely applied decision-making method. However, the number of requests for admission made at a time of extreme scarcity of ICU beds dramatically increased.

It has been shown that in case of shortage of ICU beds, the criteria for patient selection are modified, patients being more frequently considered as necessitating mainly palliative comfort care [7, 8]. It is also necessary to underline the increased risk of mortality for patients who cannot be admitted to ICU due to lack of beds, whatever the secondary course adopted: delayed admission, transfer to another distant unit or admission to a less specialized unit [9].

Faced with a massive influx of patients and extreme scarcity of ICU beds, the theoretical risk of “sacrificing the most vulnerable patients” shakes our ethical convictions. Herein, a triage plan with ethical justifications (Table 1) has been proposed to maximize benefit for the greatest number of people [3, 4, 10, 11]. Were the plan to be applied, utilitarian ethics would take precedence over individual ethics and employ the means least restrictive to individual liberty in view of accomplishing the public health goal. In other words, an unprecedentedly dramatic experience has taken place in which, due to compressed temporality, exacerbated emotional factors and massive influx of patients, a choice in the sorting cursor is made to the detriment of a reasoned strategy. Such a situation is likely to contradict our caregiving-based ethical values [12]. Indeed, in addition to the elements linked to the lack of available beds, several factors in the decision-making process were sources of concern: reduction of the minimum time necessary to make such occasionally “life-or-death” decisions, decrease due to containment measures in the essential time to be spent with relatives and pressure from the continuous flow of arriving ICU patients.

**Table 1:** Ethical values to justify priorities for critically ill patients supported

Prioritize those most likely to survive the current illness
Prioritize those most likely to live the longest after recovery (considering comorbid conditions)
Prioritize those who have lived fewer life stages
Prioritize those who have a particular narrow social utility to others in a pandemic
Prioritize the worst off (sickest or youngest)
First come, first served
Lottery

### Adapted prioritization strategies for triage

In parallel with war medicine or disaster situations, prioritization strategies have been proposed [13, 14]. Although such prioritization is not supposed to be opposed to the ethical issues of ICU access, in a specific epidemic situation this approach is nevertheless in conflict with our principles insofar as it allows utilitarian ethics to take precedence over ethics based on personhood. In this strategy, doing the greatest good for the greatest number may be inadequate insofar as it ignores other ethically relevant considerations. Among the ethical principles (Table 1), prediction of number of years to live is posited as the priority selection criterion, which means that the youngest individuals should receive priority, thereby applying the life-cycle principle in allocation decisions [15]. However, this appears to be only the least bad of existing or proposed justifications.

Decision trees have been proposed and simplified specific criteria have been requested, so to shorten the previously implemented regulatory period; this is in contradiction with a recommended practice, which privileges clinical contact with the patient. A simple score integrating the SOFA score and the estimate of a probability of death at 1 or 5 years has been used, leading to the creation of a three-grade priority standard [3, 15]. Although numerous studies have demonstrated the relevance of such scores on an overall population scale, their lack of sensitivity or specificity at the individual level has been repeatedly underlined [16–18]. Indeed, the crude AUROC for SOFA score predicting in-hospital mortality is only 0.753, leaving one out every four patients with an inappropriate decision [19]. Similarly, the ability to predict a given patient’s life expectancy or risk of mortality at 1 or 5 years is generally poor. When applied, such strategies must assume “mistake of prophecy” and the eventual sacrifice of wrongly predicted patients. Similarly, age becomes a potentially easy operational cursor, which we do not know how to place rationally [20]. However, whatever the angle of attack, we can only make our choices using ethically flawed approaches. Thus, shared recommendations including an admission decision-making checklist incorporating frailty score, comorbidities and, quality of life evaluation (Table 2), have been developed and published on Covid-crisis websites [21, 22] helping intensivists to make such decisions. To conclude, rather than promoting unrefined and imprecise outcome prediction, a pragmatic multimodal approach taking into account frailty score and, comorbidity indices while leaving room for physician judgment should be considered as the best possible [4].

**Table 2:** Ten elements that should be theoretically considered for decision to admit or not a patient in ICU

Patient
Expresses wishes (advanced directives, patient’s healthcare proxy or family)
Prognostic of acute disease and expected treatment benefits
Potentially fatal advanced chronic diseases (comorbidities)
Bad quality of life (before or estimated after the ICU stay)
Frailty score
Family or proxy
Information
Witness of patient’s willing
Collegial decision-making procedure (physicians and other healthcare professionals)
External consultant having relevant expertise (general practitioner, referral specialist, intensivist from another unit)
Recording in the patient’s medical file

As another application of the societal concept, it has been proposed to prioritize for ICU care the caregivers who have become critically ill, not due to their intrinsic quality or for so as to “reward” them, but rather for the possibility, once they are cured, of being returning to the operational caregiving circuit [3]. This raises at least two issues: first, the illusion of a rapid return to the caregiver circuit after resuscitation care for a severe form of the disease [23], and second, the choice of target actors for such prioritization. This appears to be an insoluble brain teaser: why not prioritize other societal actors who may favor the fight against pandemic such as researchers or other professionals helping to maintain the balance of our society in times of acute crisis? And with respect to the ethical principle of distributive justice, how is one to say that one life is worth more than another? Moreover, utilitarian theories of emergency ICU bed allocation have been criticized in the theoretical literature, especially on the ground of inequity in application of criteria that may disadvantage existing vulnerable populations [24].

### Creation of “Neo-ICUs”: ICU outside the walls

One solution to overcome the shortage of ICU beds during a pandemic is to quickly set up new ICUs. This requires available rooms in the hospital or the rapid construction of new units, as has been done in China. This option effectively increased the number of ICU beds by almost 100% in several countries and facilitated on-the-spot admission of large number of patients requiring mechanical ventilation. It was rendered possible by the dedication of volunteer health care workers (HCWs) having agreed to work in a new and singularly stressful environment. However, this option has been associated with a significant risk of reduced quality of care for several reasons associated with the difficulties in meeting nationwide standards for critical care facilities in this type of emergency context. First, rooms converted from intermediate care units or post-operative recovery rooms are not adequately designed for the all the equipment and organization required in critical care. Second, volunteer HCWs recruited to work in ICUs may not adequately be trained for specific and sophisticated ICU work despite the hastily improvised teaching sessions or “crash courses” organized to help them learn. Along with the risk of decreased skill level, insufficient training of these HCWs increases the burden of work [25]. Third, in the context of a pandemic, highly sophisticated devices, especially ventilators, are frequently lacking. This leads to use of inappropriate devices for the complex care of severe ARDS patients. To sum up, while the possibility of quickly setting up “Neo-ICUs” permits admission of a large number of very severe critically ill patients, it also entail a possible risk of downgraded quality level of care and subsequent impaired prognosis, as shown in other situations [26, 27]. Additionally, this type of organization may imply distributive inequality, with access to ICU facilities of heterogeneous efficiency and with a selection criterion that would be close to *first come*—*first served*, which could become *first come*—*best served*.

### Transfers of ICU patients towards distant ICUs with beds available

Epidemic intensity and ICU bed availability were reported to vary strongly across countries and also within regions in a single country. To mitigate these “geographic” inequalities, patient transfers from regions with dramatic shortages of ICU beds to areas less affected by the outbreak and with a large amount of available ICU beds along with including optimal material and ICU staff, have been implemented.

These transfers require aircrafts, helicopters or trains that have been sophistically adapted to the care of critically ill patients and necessitate the involvement of a large number of dedicated physicians and nurses to ensure adequate organization and optimal patient safety. Notwithstanding its complexity, in order to be efficient this transfer strategy should be organized within a short period of time and should allow the transfer of a significant number of patients. It is associated with increased costs that should not be charged to the patient or his or her relatives. The first ethical issue surrounding such transfers is related to the benefit/risk balance. For the patient, the benefit of being in the hands of highly qualified teams is counterbalanced by the risk of clinical worsening during transfer. During patient selection, close attention should be paid to severity status: not too severe (transfer would be too risky), and not too well (to avoid unnecessary transfer). While informed patient consent should theoretically be part of the decision, most of the transferred patients were unconscious and unable to approve such a transfer, thereby ruling out the autonomy principle. Informed consent was consequently obtained from their next of kin (patients whose next of kin refused were not transferred). A second ethical issue concerns the ICU departments accepting patients from a distant region and possibly aggravating the risk of a suddenly increased epidemic wave in their own area. Indeed, Covid 19 pandemic experience has shown that we did not have efficient predictive tools to precisely anticipate the kinetics of ICU bed requirements. Finally, such transfers may be associated with increased suffering and psychological trauma for the relatives. Indeed, long distance and limitation of travels for epidemic control will strongly impede if not altogether rule out the presence of relatives at the patient’s bedside and prevent adequate communication between them. This could exacerbate pain for the families, especially if specific communications are not developed (see below).

### Separating the caregivers in charge of the patient and the triage team

It has been proposed to relieve the ICU teams in charge of patient care of the responsibility of admission or non-admission decisions and to entrust this work to a dedicated triage team headed by a triage officer [15, 28]. The advantage of this approach is that it relieves the healthcare team of the emotional impact of a potentially painful ethical dilemma [3]. However, the composition of these triage teams must be specified. Mentions of volunteers, leaders recognized by their peers and by the medical community have been put forward [28]. It should no doubt be specified that the triage leaders will be intensivists recognized for their ethical sensitivity, and an overly “military” strategy should be scrupulously avoided [29]. If not, the potentially protective role of independent triage teams can be a source of additional injury for caregivers, disappointed with their patient’s unfavorable outcome and even blamed for an unshared therapeutic cessation decision or dehumanization of care [30].

### Modification in the decision-making process to withhold or withdraw life support treatments due to the epidemic context

It has been suggested that patient severity assessments be intensified during their progress in ICU stay, so that the withdrawal of one patient’s mechanical ventilation can benefit another patient [3]. In this way, withdrawal of artificial ventilation might be decided when the improvement is not fast enough, while hopes of survival may persist. Similarly to the triage team, it has been proposed to use triage committee to buffer clinician from potential harm [1, 24]. Again, the risk of ethical drift must be emphasized. Despite an influx of patients and lack of beds, it does not seem ethically acceptable to lose a chance for patients for whom treatment does not seem to be unreasonable obstinacy. Moreover, the appreciable time taken to make these decisions is an element that risks being called into question during an epidemic emergency. Finally, under the pretext of risk of contamination and need for confinement, exchanges with relatives to share final decisions could be reduced if not eliminated, a factor entering once again into contradiction with basic ethical concepts.

It must be admitted that in a crisis situation with an unprecedented influx of patients in ICU, no single strategy fully corresponds to our ethical values. Whatever the approach adopted, imbalance between societal and individual ethics leads to unsolvable discomforts that caregivers will have to overcome. In other words intensivists would have to consider their own tension between utilitarianism (making ICU beds available rapidly, potentially sacrificing patients without rapid improvement for new admissions) and virtue (accept to prolong ICU stay for an ICU patient even if there is no bed available to admit another patient) ethics. Fortunately, the formalized strategies of ethical reflection associated with decision-making for withdrawal of life support therapies have long since been part and parcel of routine practice, leaving the ultimate choice of decision up to the intensivist. The heterogeneity in EOL-decision-making is probably huge across hospitals and ICU. Postponed decision-making or even paralysis at EOL may have created excess in mortality due to shortage of ICU beds. Nevertheless, confidence should be given to ICU teams to manage the eventual withdrawal of life support decision through a bedside decision-making process taking into account the exceptional difficulties linked to the epidemic situation.

### Setting priorities in ICU resources during a pandemic: the role of public opinion

Since discrepancies may exist between experts’ ethical recommendations and public perception, general public opinion has been investigated based on the basis of deliberative democracy [13, 24]. A 228-participant panel placed in a simulated context of a severe influenza pandemic favored ethical principles of saving the most lives (surviving current illness) and saving the most life-years (living longer) over a first come first serve scenario [13]. However, a significant number of participants were opposed to the idea of ventilator reallocation [13]. In this study, subgroup differences associated with age or ethnicity of the participants were pointed out [13]. In another survey, the pragmatic constraints imposed by an assumption of extreme scarcity were not accepted by the Canadian participants, who expressed difficulties in making priority-setting decisions because these were perceived as psychologically burdensome, no-win situations [24]. Transparent communication is also important during such a crisis so as to allow public opinion to be able to better understand place the decisions of ICU teams.

### Family visits and family-centered care

The COVID-19 epidemic is a threat to family-centered care in ICUs. During the 1st weeks of the epidemic, visits were prohibited to ensure that relatives did not contaminate other family members, patients, or healthcare professionals. Family members could no longer be at the patient’s bedside and the ICU team was unable to propose structured communication and support to family members. Involvement in decision-making was compromised, and it was felt that this situation was harmful both for patients and family members.

Indeed, over the last decade, research has shown that Post-ICU syndrome (PICS-F) [31] in family members is a cause of major concern. The major risk factors for PICS-F are poor communication with an ICU team, being in a decision-making role, low educational level, and having a loved one who died or was close to death. Indeed, many studies have shown that communication with caregivers is one of the most highly valued aspects of care and that impacts-on family members’ experience during and after the patient’s stay, including in the aftermath of the patient’s death [32, 33]. Communication perceived as inconsistent, unsatisfactory or uncomforting is associated with higher risk of post-ICU burden [34]. Risk of PTSD-related symptoms increases when relatives, both non-bereaved and bereaved, feel that the information given is incomplete [35].

After death in the ICU, bereaved family members are at high risk of presenting symptoms that negatively affect their quality of life, such as anxiety, depression, PTSD symptoms [35, 36] and complicated grief [37]. Interestingly, family members who witness a relative of theirs suffering from dyspnea are at higher risk of developing PTSD-related symptoms and those who are not able to say goodbye to relative of theirs are at higher risk of developing complicated grief symptoms [37].

In the context of the Covid-19 pandemic, risk factors for developing post-ICU burden are numerous, thereby increasing exposure to anxiety, depression, PTSD and complicated grief. As said in the New York Times, “*Of all the ways the coronavirus pandemic has undermined the conventions of normal life, perhaps none is as cruel as the separation of seriously ill patients and their loved ones, now mandated at hospitals around the world*” [38].

Faced with these various difficulties and risks, recommendations have been published regarding communication with family members in this specific context. First, patients and family members should receive clear explanations, both directly (over the phone or when present) and on institutional websites, concerning the imposed restrictive policies: it is important that they understand why they cannot visit their loved one [39]. In other words, the restriction must have meaning. Second, ICU teams are encouraged to proactively schedule routine telephone calls with family members to maintain continuity of communication [40]. The calls must follow a plan so that family members know when to expect contact. The phone calls will not only address the patient’s health status, but also provide reassurance regarding comfort and dignity [41]. Conversations are important to help the ICU team better understand the patient as a person (values, advance directives, etc.) and to help family members think about possible difficult decisions. In this context, goal-concordant care is particularly important and ICU teams must strive to avoid intensive life-sustaining treatments that would be unwanted by patients [42]. On a parallel track, strategies to reinforce communication between the patient and the family have been developed. ICU teams should encourage patient and family to call, text, and videoconference with each other as often as wanted [39]. They may also help the patient and family members record and send audios, videos, or written messages to one another. If the patient is unconscious, the ICU team can print written messages or family photos and stick them in a diary that can then be given to the patient. Staying in touch is vital, both for the patient and for the family members.

Moreover, many ICU teams have made visitation policies more flexible. These units have adapted themselves to the influx of patients while respecting a predetermined protocol. The visitor must have a dedicated time appointment and wait in a room where he/she may not meet other visitors. Instructions on hygiene are given by the nurses. Psychological support for each visit and follow-up calls by the ICU psychologist are also recommended. Visiting a loved one in intensive care is very upsetting in the best of times, but when in addition one has been separated for days, perhaps weeks, there is also all the emotional pressure of a long-awaited reunion.

### End of life

In end-of-life (EOL) situations, the ICU team must avoid depriving family members of the opportunity to say goodbye to the patient [43]. If visitation is usually forbidden in the ICU, it should be made possible in an EOL situation. If the family cannot or does not want to come to the ICU, letting him/her speak to the patient one last time over the phone is important. Family members need to prepare for bereavement, meaning they must understand what is happening: end-of-life family conferences should be organized, remotely if needed [40]. Honest conversations are important, as helping family members prepare for death is an important part of anticipatory grief [44]. Not being prepared is associated with increased risk of complicated grief.

When possible, respecting the family’s wishes is particularly important in a context where the grief process may be more complex as families are unable to see their loved one’s body, to physically share their emotions with other relatives and, sometimes even to attend their loved one’s burial.

### Health care workers’ psychological disorders

In the current pandemic, sources of psychological disorders for HCWs are multiple. They are affected by distress similar to than the general population regarding the effects of lockdown and containment, the risk of personal or families’ and friends’ illnesses, the uncertainty about pandemic duration and, the lack of effective specific treatment. This dearth of knowledge has given rise to a great deal of contradictory information that has forced health care professionals to constantly readapt and to cope with the experience of powerlessness and personal ineffectiveness [5, 45], and they also experience “front line” specific factors [46]. The factors include extended workloads, feelings of powerlessness when trying to contain the large number of patients, concerns about the suffering and potential poor outcomes of their patients, preoccupations about potential shortages of intensive care resources (including personal protective equipment), the fear of transmitting the disease to their loved ones, and apprehension about possible involvement in ethically difficult resource allocation decision-making. This situation has created a high level of uncertainty and insecurity that constitutes a risk to the mental health of caregivers [47, 48]. To date a few studies have reported a quantification of symptoms amongst HCWs. All of them have shown an increase in psychological disorders compared with different control groups providing no direct care to patients: [4, 36–38, 49]. Fear was more frequent than anxiety and depression with incidences varying from 71, 25, 12% to 73, 44, 50%, respectively [50, 51]. Assessments by other scales confirmed two-thirds of mental health disorders, especially in young women [52]. Sleep disorders were also reported [53]. In some countries, e.g., in Italy or in France, healthcare workers are applauded by the population each evening at 8 pm. Societal reward and “glorification” [54] of the caring function appears to be a protective factor in the short term [48] and in his first address to the nation, the President of France, Emmanuel Macron, called healthcare workers “the heroes with a white coat”. It may be dangerous for healthcare workers to fall into this trap. Altruism has long since been recognized as a core value of this profession. Moreover, a hero must keep silence about his feelings, a factor which is known to favor burnout [55].

Insecurity and uncertainty are reflected not only at an individual level, but also at a collective level. The COVID-19 epidemic requires reinforcement of the ICU teams with new staff members or even reorganization of the unit, weakening the reference points and trust within the team. This context creates a feeling of vulnerability and loss of control for professionals [56, 57]. A lack of interaction between caregivers and families induces a feeling of exclusion and even a significant emotional burden when patients die, highlighted in certain cases by a feeling of guilt [30]. Psychological support has been set up for caregivers, as many hospitals have initiated telephone hotlines, psychologists within units, relaxation sessions, meditation, discussion groups, and optimization techniques. These responses should ideally vary according to the phase of the pandemic [58]. At the early phase, the best way to prevent psychological disorders is to acknowledge staffers’ work by providing adequate human resources and material supplies [59]. Both frequency and transparency in hospital communication likewise play a key role [58, 60]. Concrete measures to set up rest areas, to facilitate the logistics of meals, daily life, and the possibility of having leisure and relaxation time are optimally appropriate to the needs of the caregivers during the crisis. At this stage, this type of collective support could be more effective than individual support. However, individual assessment of mental health may later become relevant. In a study in Wuhan, the most valued psychological resources consisted in social media (50%) and psychological guidance books (36%) [52]. Requests for therapist-driven video calls or consultations were less frequent (17%) and rose the question of their availability, given the large number of affected HCWs [52]. Similarly, a form of reluctancy, or even an absence of solicitation of the listening units in times of health crisis has been reported [47, 48].

## Conclusions

To overcome the Covid-19 pandemic, in many places throughout the world, new resources were developed in a short period of time, dramatically increasing the number of ICU beds allowing admission of a huge number of critically ill patients. The massive patient influx highlighted numerous ethical concerns that ICU caregivers are likely to face. Some models have proposed ethical justifications to difficult decision-making, usually based on deontological (*or societal*) rather than individual ethics. We wished to draw attention to the risk of taking refuge behind ethical alibis notwithstanding the fact that the specific pandemic context there is no single satisfactory solution. In such a situation each option is associated with its own strengths and weaknesses, and intensivists should make their choices in full awareness of intractable ethical dilemmas. In many circumstances, caregivers have no choice but to adopt less than perfect solutions even though the price to be paid consists in undermining patients’, relatives’ and caregivers’ psychological well-being. Lessons should be learnt from this experience and ethical reflections should be developed in order to anticipate a potential new pandemic in the close or more distant future.

## Data Availability

Not applicable.

## Abbreviations

EOL: End-of-life
HCWs: Healthcare workers
ICU: Intensive care unit
